# Brain Metabolic Dysfunction in Early Neuropsychiatric Symptoms of Dementia

**DOI:** 10.3389/fphar.2019.01398

**Published:** 2019-11-22

**Authors:** Kok Pin Ng, Hui Jin Chiew, Pedro Rosa-Neto, Nagaendran Kandiah, Zahinoor Ismail, Serge Gauthier

**Affiliations:** ^1^Department of Neurology, National Neuroscience Institute, Singapore, Singapore; ^2^The McGill University Research Centre for Studies in Aging, Montreal, QC, Canada; ^3^Duke-NUS Medical School, Singapore, Singapore; ^4^Departments of Psychiatry, Clinical Neurosciences, and Community Health Sciences, Hotchkiss Brain Institute and O'Brien Institute for Public Health, University of Calgary, Calgary, AB, Canada

**Keywords:** neuropsychiatric symptoms, Alzheimer's disease, metabolic dysfunction, [^18^F]fluorodeoxyglucose PET, mild behavioral impairment

## Abstract

Neuropsychiatric symptoms (NPS) including behavioral and psychiatric symptoms are common in the dementia stages of Alzheimer's disease (AD) and are associated with poorer outcomes in cognition, functional states, quality of life, and accelerated progression to severe dementia or death. NPS are also increasingly observed in the mild cognitive impairment stage of AD and may predict incipient dementia. As such, there is an emerging conceptual framework, which support NPS as early non-cognitive symptoms of dementia. [^18^F]fluorodeoxyglucosepositron emission tomography is a technique that is sensitive in detecting resting metabolism associated with NPS in neuropsychiatric conditions, and there is a growing body of literature evaluating the role of NPS as early indicators of brain metabolic dysfunctions in AD. In this mini-review, we examine the frequency and associations of NPS with metabolic dysfunction in the AD continuum, including preclinical, prodromal, and dementia stages of AD. We will also present the validated neurobehavioral syndrome, mild behavioral impairment describes the later life emergence of sustained NPS as an at-risk state for incident cognitive decline and dementia, and an early presentation of neurodegenerative diseases in some. Lastly, we will discuss future directions in the field so as to better understand the neurobiological basis of NPS in the early stages of the AD continuum, and their role in predicting AD pathophysiological progression and incident dementia.

## Introduction

Neuropsychiatric symptoms (NPS) are non-cognitive symptoms common in the Alzheimer's disease (AD) continuum (Lyketsos et al., 2011; Brodaty et al., 2015; Vik-Mo et al., 2018), associated with poorer cognitive, functional, and quality of life outcomes, and accelerated progression to severe dementia (Teng et al., 2007; Karttunen et al., 2011; Fischer et al., 2012; Peters et al., 2015). An emerging conceptual framework proposes NPS as early clinical manifestations in the preclinical and mild cognitive impairment (MCI) stages of AD, better predicting progression to dementia than those without NPS (Donovan et al., 2014; Geda et al., 2014; Burhanullah et al., 2019;Ruthirakuhan et al., 2019; Wise et al., 2019). Therefore, studies of NPS in the early stage of AD are paramount, given that insight into the underlying neurobiology of early NPS may enable targeted interventions to improve clinical outcomes (Mortby et al., 2018a).

[^18^F]fluorodeoxyglucose ([^18^F]FDG) positron emission tomography (PET) measures cerebral glucose metabolic rate (CMRglc) changes and AD studies using [^18^F]FDG PET have demonstrated correlations between CMRglc reduction in the parietotemporal, posterior cingulate (PCC), and medial temporal and/or frontal cortices and synaptic dysfunction (Jack et al., 2016). Progressive CMRglc reductions occur years prior to clinical symptoms in patients with pathologically verified AD (Mosconi et al., 2009) and the degree of CMRglc reduction relates to disease severity (Furst et al., 2012). Studies in depression (Kennedy et al., 2001) and schizophrenia also demonstrate regional cerebral metabolic dysfunction correlating with psychiatric symptoms. Therefore, [^18^F]FDG PET constitutes a valuable neuroimaging modality to investigate the relationship between NPS and metabolic changes in early stages of AD.

In this mini-review, we will examine the associations of NPS and metabolic dysfunction in the AD continuum, including preclinical, prodromal, and dementia stages of AD. We will also discuss a recently proposed diagnostic construct, mild behavioral impairment (MBI), that determines the emergence of NPS in later-life as an early manifestation of neurodegenerative diseases.

## Frequency of Neuropsychiatric Symptoms in AD

NPS are commonly observed across the AD continuum. In two population-based studies, 61%–75% of demented participants exhibited one or more NPS in the past month, with apathy, depression, and agitation/aggression being most common (Lyketsos et al., 2000; Lyketsos et al., 2002). A systematic review and population studies also show that behavioral abnormalities are observed in 35%–75% of MCI patients, with depression, apathy, anxiety, and irritability being most common (Lyketsos et al., 2002; Apostolova and Cummings, 2008; Geda et al., 2008). Another systematic review and meta-analysis found the prevalence of depression in MCI to be 32%, but higher in clinical (40%) vs. community (25%) settings, emphasizing the clinical significance of NPS (Ismail et al., 2017b). In the Dominantly Inherited Alzheimer Network (DIAN), early behavioral changes such as depression, apathy, disinhibition, irritability, sleep changes, and agitation are also more common in mildly symptomatic familial AD mutation carriers relative to non-carriers (Ringman et al., 2015).

NPS in cognitively normal individuals predict subsequent cognitive decline (Burhanullah et al., 2019). In a prospective cohort study over a median of 5.0 years, baseline NPS in cognitively normal persons also increased the risk of incident MCI (Geda et al., 2014). However, in DIAN, depressive symptoms were less common in cognitively asymptomatic mutation carriers than in non-carriers and the odds of experiencing at least one behavioral symptom in asymptomatic carriers was lower than in non-carriers (Ringman et al., 2015).

## Methods

A PUBMED search was conducted using the keywords "FDG," "fluorodeoxyglucose," "PET," "positron emission tomography," "metabolism," "Alzheimer," "dementia," "mild cognitive impairment," "predementia," "neuropsychiatric," and "behavior" in January 2019. A total of 5243 English language articles were found. Fifty articles reporting on metabolic dysfunction in dementia and cognitive impairment were identified, of which 32 studies reporting on non-AD dementia were excluded. Of the remaining 18 articles, 1 was excluded as the diagnosis of AD could not be separated from other causes of dementia. The remaining 17 articles (12 in AD, 5 in MCI or preclinical AD) were discussed here.

## Metabolic Dysfunction and NPS

It has been proposed that NPS in AD may cluster into specific subsyndromes and share similar clinical trajectories (Aalten et al., 2007; Canevelli et al., 2013; Nowrangi et al., 2015; Ballarini et al., 2016). In a large European cohort of more than 2000 AD subjects, four subsyndromes were identified based on the Neuropsychiatric Inventory (NPI): apathy, affective, hyperactivity, and psychosis (Aalten et al., 2007). Currently, the neurobiological basis of neuropsychiatric subsyndromes, especially in the early stages of AD remained poorly understood, and each subsyndrome may have distinct underlying neuroanatomical and neurobiological correlates (Nowrangi et al., 2015; Ballarini et al., 2016).

In this section, we present and discuss the evidence for metabolic dysfunction in each neuropsychiatric subsyndrome across the AD continuum. Where data are available, we emphasize preclinical and prodromal stages of AD (**Tables 1** and **2**).

**Table 1:** Summary of metabolic dysfunction in AD dementia subjects with NPS by subsyndromes.

NPS Subsyndrome	Study	NPS	Dementia	Number of subjects	Number of subjects with NPS	Findings
Affective	Sultzer et al., 1995	Anxiety/ depression	AD (mean MMSE 18.6)	21	Not available	Parietal lobe hypometabolism*
	Hirono et al., 1998a	Depression	Mild to moderate AD	53	16 (35.9%)	Bilateral superior frontal and left anterior cingulate cortex (ACC) hypometabolism
	Holthoff et al., 2005	Depression	Mild to moderate AD	53	10 (18.9%)	Left superior frontal and prefrontal cortex hypometabolism
	Lee et al., 2006	Depression	Mild AD	12	6 (50%)	Right superior frontal gyrus hypometabolism
	Hashimoto et al., 2006	Anxiety	AD (mean MMSE 19.6)	41	19 (46.3%)	Left superior temporal gyrus, entorhinal cortex, and parahippocampal region hypometabolism
	Ballarini et al., 2016^#^	–	EOAD (mean MMSE 20.78)	27	17 (63%)	Bilateral ACC and superior frontal gyrus (extending to supplementary motor area) hypermetabolism
Apathetic	Holthoff et al., 2005	Apathy	Mild to moderate AD	53	17 (32.1%)	Bilateral orbitofrontal cortex (OFC) hypometabolism
	Marshall et al., 2007	Apathy	AD (mean MMSE 19.6)	41	14 (34.1%)	Bilateral ACC and medial OFC hypometabolism
	Ballarini et al., 2016^#^	–	EOAD (mean MMSE 20.78)	27	20 (74.1%)	Bilateral middle frontal gyri and OFC hypometabolism
Hyperactivity	Sultzer et al., 1995	Agitation/ disinhibition	AD (mean MMSE 18.6)	21	Not available	Temporal lobe hypometabolism*
	Ballarini et al., 2016^#^	–	EOAD (mean MMSE 20.78)	27	19 (70.4%)	Left insula, superior frontal, anterior cingulate gyrus, temporal pole, precentral gyrus, and right inferior frontal gyrus hypermetabolism
	Weissberger et al., 2017	Agitation	AD (mean MMSE 19.3)			Bilateral posterior cingulate, right middle temporal gyrus, and right frontal cortex hypometabolism
Psychotic	Sultzer et al., 1995	Psychosis	AD (mean MMSE 18.6)	21	Not available	Frontal lobe hypometabolism*
	Mentis et al., 1995	Delusions of misidentification	Mild to moderate AD	24	9 (37.5%)	Bilateral OFC and cingulate hypometabolism Bilateral sensory association cortex hypermetabolism
	Hirono et al., 1998b	Delusions	Mild to moderate AD	65	26 (40%)	Left medial occipital region Left inferior temporal gyrus hypermetabolism
	Sultzer et al., 2003	Delusions	AD (mean MMSE 16.5)	25	76%	Right superior dorsolateral and inferior frontal hypometabolism. Right lateral orbifrontal hypermetabolism.
	Sultzer et al., 2014	Delusions	AD (mean MMSE 19.3)	88	28 (31.8%)	Right lateral frontal, orbitofrontal, and bilateral temporal cortex hypometabolism

*Derived from mean [^18^F] FDG-PET metabolism from right and left hemispheres.

^#^NPI symptoms clustered into subsyndromes: affective (anxiety and depression); apathetic (apathy and eating and appetite changes); hyperactivity (agitation/aggression, irritability, euphoria/elation, aberrant motor behavior, and disinhibition); psychotic (delusions, hallucinations and night-time sleep disturbances). Breakdown not available.

**Table 2:** Metabolic dysfunction and NPS in preclinical AD and MCI subjects.

Studies	NPS	Number of subjects	Subjects with NPS	Findings
Marshall et al., 2013	Apathy	24 amnestic MCI	Not available	No significant association
Delrieu et al., 2014	Apathy	65 amnestic MCI	11 (16.9%)	Bilateral posterior cingulate (PCC) hypometabolism
Gatchel et al., 2017	Apathy	402 subjects: -203 amnestic MCI -104 cognitively normal -95 mild AD	73 (18.2%)	Bilateral PCC hypometabolism
Lee et al., 2010	Depression	36 amnestic MCI	18 (50%)	Right superior frontal gyrus hypometabolism
Brendel et al., 2015	Depression	209 Aß +ve MCI 165 Aß -ve MCI	65 (31.1%) 41 (24.8%)	Right superior frontal, left middle frontal and left fusiform gyri hypermetabolism in Aß +ve subjects

### Apathetic Subsyndrome

The apathetic subsyndrome consists of apathy, eating abnormalities, and aberrant motor behavior. However, existing literature consist of [^18^F]FDG PET studies either in the apathetic subsyndrome or apathy only. On the whole, there is correlation between apathy and hypometabolism in the orbitofrontal cortex (OFC) and cingulate cortex in dementia subjects; while in MCI, apathy appears to be correlated with an AD-specific pattern of hypometabolism in the PCC.

In a cohort of 53 AD patients with mean disease duration of 28.7 months and Mini-Mental State Examination (MMSE) score of 22.5, apathy was associated with hypometabolism in the left OFC (Holthoff et al., 2005). In 41 AD patients, hypometabolism in bilateral ACC and bilateral medial OFC were reported (Marshall et al., 2007). Ballarini et al. examined the associations between regional metabolism, functional connectivity and neuropsychiatric subsyndrome clusters in early onset AD (EOAD). In 51 EOAD subjects, 27 had NPS, of which apathetic subsyndrome was the most common (74%). Hypometabolism was found in bilateral middle orbitofrontal and middle frontal gyri of subjects with the apathetic subsyndrome (Ballarini et al., 2016).

There are fewer [^18^F]FDG PET studies in prodromal AD. A small study of 24 MCI subjects showed no significant association between apathy and regional glucose metabolism (Marshall et al., 2013). A larger study of 65 MCI individuals from the Alzheimer's Disease Neuroimaging Initiative (ADNI) database showed an AD-specific pattern of PCC hypometabolism in MCI subjects with apathy (Delrieu et al., 2014). This was corroborated by a subsequent ADNI study including 422 cognitively normal, MCI, and early dementia subjects, demonstrating correlation between PCC hypometabolism and higher apathy scores (Gatchel et al., 2017). Baseline hypometabolism of the supramarginal gyrus was also found to predict the increase of apathy over time (Gatchel et al., 2017).

In AD dementia, the association between apathy and hypometabolism in the OFC and anterior cingulate cortex (ACC) is consistent with their role in recognition of salient stimuli, reward-based decision-making, drive, and motivation (Holthoff et al., 2005; Wallis, 2007; Kouneiher et al., 2009). This is supported by a number of studies using other imaging modalities such as magnetic resonance imaging (MRI), diffusion tensor imaging and single-photon emission computed tomography (Stella et al., 2014). Indeed, the ACC has been recognized as a key node in the salience network (SN) (Seeley et al., 2007; Menon, 2015).

In MCI with apathy, the finding of PCC hypometabolism mirrors the early metabolic dysfunction characteristically seen in amnestic MCI subjects reflecting underlying AD pathology (Drzezga et al., 2003; Nestor et al., 2003). The reason for sparing of frontal lobe metabolism is less certain. Firstly, the degree of apathy may be below the threshold for detection of hypometabolism in the OFC and ACC (Delrieu et al., 2014). Secondly, the frontal and parietal regions are interconnected, and dysfunction in one or more parts of the network may give rise to apathy (Gatchel et al., 2017).

### Affective Subsyndrome

The affective subsyndrome comprises anxiety—a "positive" symptom—and depression—a "negative" symptom. [^18^F]FDG PET studies have shown metabolic dysfunction in the superior frontal and ACC of subjects with affective subsyndrome in various stages of dementia, though studies in MCI are lacking (Sultzer et al., 1995; Hirono et al., 1998a; Holthoff et al., 2005; Hashimoto et al., 2006; Lee et al., 2010; Brendel et al., 2015; Ballarini et al., 2016). Two studies examined anxiety and depressive symptoms together as a subsyndrome in moderate AD (Sultzer et al., 1995; Ballarini et al., 2016). An early study showed reduced metabolism in the parietal lobes of subjects with moderate AD with anxiety/depression (mean MMSE 18.6, mean disease duration of 4.2 years) (Sultzer et al., 1995). In 17 EOAD subjects with affective subsyndrome, increased metabolism in the superior frontal gyri and ACC was demonstrated (Ballarini et al., 2016). These findings reflect the important role of the ACC in the SN, which mediates the "top-down" selection of significant emotional and sensory stimuli, directing attention and influencing goal-directed behavior (Seeley et al., 2007). More specifically, the ACC is involved in response selection and conflict monitoring (Menon, 2015). Therefore, increase in nodal activity in the ACC may lead to aberrant emotional responses to salience, especially the "positive" symptom of anxiety (Zhou and Seeley, 2014).

Furthermore, in a cross-sectional study of cognitively normal persons aged ≥ 70 years from the Mayo Clinic Study of Aging, depressive and anxiety symptoms were associated with decreased [^18^F]FDG PET uptake in AD-related regions (Krell-Roesch et al., 2016), suggesting that NPS may play an important role in addition to the current biomarker-based investigations in presymptomatic AD.

Depression as an individual symptom has been studied in MCI and mild AD, demonstrating an association with abnormal glucose metabolism predominantly in the frontal lobes. In earlier studies, depressive symptoms in AD correlated with hypometabolism especially in the superior frontal gyri (Hirono et al., 1998a; Holthoff et al., 2005; Lee et al., 2010). However, a recent [^18^F]FDG PET study of 371 MCI ADNI subjects showed hypermetabolism in the right superior frontal, left middle frontal, and left fusiform gyri in amyloid-positive MCI subjects (Brendel et al., 2015), consistent with the aforementioned findings in EOAD subjects with the affective subsyndrome (Ballarini et al., 2016). These findings are concordant with those in non-demented subjects with late-life depression, where hypermetabolism in the superior frontal gyri is correlated with severity of depression (Smith et al., 2009). The role of the superior frontal gyri in depression, and its relation to amyloid pathology warrants further study.

### Hyperactivity Subsyndrome

Data of metabolic dysfunction and hyperactivity subsyndrome (agitation/aggression, euphoria, disinhibition, and irritability) in AD is limited, especially in the preclinical or prodromal stages. Significant correlation between the agitation/disinhibition factor score of the Neurobehavioral Rating Scale (NRS) and hypometabolism in the frontal and temporal lobes in 21 AD subjects have been demonstrated (Sultzer et al., 1995). In an EOAD cohort with mean disease duration of 3.18 years and MMSE 20.7, hypermetabolism in the left insula, superior frontal gyrus, temporal pole and precentral gyrus, the ACC, and the right inferior frontal gyrus were found in 19 subjects with the hyperactivity subsyndrome (Ballarini et al., 2016). This contrasts with a recent study in 88 mild to moderate late-onset AD (LOAD) (mean age 78 years, disease duration 3.2 years, MMSE 19.3), which instead found hypometabolism in the right temporal and bilateral middle and posterior cingulate regions in subjects with agitation (Weissberger et al., 2017).

The association between hyperactivity and metabolic dysfunction in the ACC and insula is explained by their roles in the SN (Menon, 2015). In mild to moderate AD with hyperactivity, increased functional connectivity in the anterior SN was demonstrated (Balthazar et al., 2014). Studies using structural MRI in MCI and mild AD with agitation have shown greater atrophy in regions of the SN such as the ACC, insula, and amygdala (Bruen et al., 2008; Trzepacz et al., 2013). Taken together, the evidence suggests a link between neurodegeneration in AD, dysfunction in the SN, and the hyperactivity subsyndrome. The reason for the discordant findings in metabolic dysfunction in EOAD and LOAD with agitation, however, remains to be elucidated.

A recent study of preclinical sporadic AD with both amyloid and tau pathologies present showed that NPS, driven by irritability and sleep behavior domains, are linked to metabolic dysfunction within the limbic networks that are vulnerable to AD. NPS also predict subsequent hypometabolism in the PCC. These findings suggest that NPS may represent an early clinical manifestation of AD pathophysiology (Ng et al., 2017).

### Psychotic Subsyndrome

Studies on metabolic dysfunction in the psychotic subsyndrome (delusion, hallucinations, night time disturbances) are mostly in AD subjects with delusions. We are unable to find similar studies in preclinical AD or MCI, unsurprising given that these NPS are less reported in the early stages of AD (Apostolova and Cummings, 2008) and are often given psychiatric diagnoses (Fischer and Agüera-Ortiz, 2018).

Sultzer et al. first demonstrated hypometabolism in the frontal lobes in AD subjects with higher psychosis factor score on the NRS (Sultzer et al., 1995). Hirono et al. found increased left inferior temporal gyrus and decreased left medial occipital metabolism in 26 subjects with predominantly moderate-severe AD (Hirono et al., 1998b). Two subsequent studies, including a larger study of 88 subjects with mild to moderate AD, showed mainly right-lateralized findings, with hypometabolism in the right lateral, inferior and orbitofrontal cortices, as well as bilateral temporal lobes (L Sultzer et al., 2003; Sultzer et al., 2014). This is consistent with structural, perfusion, and metabolic imaging studies in AD subjects that implicate right hemispheric pathology—in particular the right frontal lobe—in the formation of delusions (Ismail et al., 2012). Right hemispheric dysfunction may cause impaired salience, self-monitoring, perceptual integration, and release of the left frontal lobe, resulting in overactivity of the default mode network (DMN) and a hyper-inferential state that predisposes to delusions (Ismail et al., 2012; Gurin and Blum, 2017).

Specific subtypes of delusions may also be associated with abnormal glucose metabolism in distinct regions of the brain. Delusional misidentification syndrome (DMS) in AD was associated with hypometabolism in bilateral paralimbic and left medial temporal regions as well as normalized hypermetabolism in the sensory association cortices (Mentis et al., 1995). This is consistent with a more recent voxel-based morphometry study showing reduced right hippocampal grey matter volume in five AD subjects with DMS, suggesting a role for medial temporal lobe dysfunction in DMS (Serra et al., 2010). Further studies clearly differentiating persecutory type and misidentification delusions are required to better understand the neurobiology of these symptoms (Ismail et al., 2011).

## Mild Behavioral Impairment

MBI is a validated neurobehavioral syndrome characterized by later life emergent and sustained NPS as an at-risk state for incident cognitive decline and dementia, and the index manifestation of dementia in some (Taragano et al., 2009; Ismail et al., 2016; Creese et al., 2019; Matsuoka et al., 2019). MBI, which may precede or co-exist with subjective cognitive decline (SCD) or MCI, represents a later-life change in behavior or personality in the domains of drive and motivation (apathy), affective regulation (mood/anxiety symptoms), impulse control (agitation, reward salience), social cognition (socially inappropriate behavior), and perception/thought content (psychotic symptoms) for ≥6 months. MBI reflects the neurobehavioral axis of pre-dementia risk states, which complements the neurocognitive axis identified by SCD and MCI. Both axes identify individuals who may have increased risk of developing dementia, and there may be a common genetic etiology for MBI and AD (Andrews et al., 2018). Importantly, MBI offers a systematic way to approach later life psychiatric symptomatology, in order to differentiate between late life psychiatric conditions for which the links to dementia are not clear (Panza et al., 2010), and later-life emergent NPS, for which the links to dementia are very clear and supported by an increasing evidence base (Rosenberg et al., 2013; Geda et al., 2014; Wise et al., 2019). A 5-year longitudinal study demonstrated this difference to be meaningful, with the MBI group having a significantly higher rate of incident dementia compared to the late-life psychiatric disorder group (Taragano et al., 2018). In many dementia clinical trials, some with NPS, especially more severe NPS, are excluded from studies. However, severity alone is insufficient to distinguish between a psychiatric condition and a potential manifestation of prodromal dementia. The age of onset and natural history of psychiatric symptomatology are the essential elements required to distinguish groups (Ismail et al., 2018). Three large observational cohorts (totaling 42,000 participants) with up to 28 years of follow-up demonstrated a link between later life emergence of psychiatric conditions, and dementia diagnosis, to be 5–11 years depending on the study, with authors of all studies suggesting that these later life psychiatric conditions may in fact have been prodromal dementia (Almeida et al., 2017; Singh-Manoux et al., 2017; Tapiainen et al., 2017)

Thus, the key to incorporation of MBI into dementia research lies in appropriate case ascertainment. In a recent study of cognitive neurology patients, MBI was present in 83.5% of MCI and 76.5% of SCD (Sheikh et al., 2018). However, MBI was diagnosed using the Neuropsychiatric Inventory Questionnaire (NPI-Q), which is a limitation, given that NPI-Q is originally designed for individuals with dementia, and has a 1-month reference range, thus not necessarily capturing the 6-month symptom duration requirement for MBI diagnosis. This short reference range can result in poor specificity, inappropriately capturing as cases with transient symptoms and reactive conditions, thus inflating frequencies. A similar analysis in a population sample of 1,377 participants with normal cognition, pre-MCI and MCI, found MBI prevalence to be 34.1% (Mortby et al., 2018b), which is also likely to be an inflated estimate. The Mild Behavioral Impairment Checklist (Ismail et al., 2017a) (MBI-C, available at http://www.MBItest.org) was thus developed, specifically as a MBI case ascertainment instrument, consistent with the new MBI criteria, and for and monitoring of MBI symptoms in pre-dementia populations. In a primary care validation study, MBI prevalence was 14.2% in MCI using a cutoff point of 6.5 and 5.8% in SCD using a cutoff of 8.5 (Mallo et al., 2018; Mallo et al., 2019). The lower and more specific prevalence estimates likely better reflects an enriched population for biomarker positivity (Lussier et al., 2019), with a greater risk for incident cognitive decline and dementia. Thus, MBI offers an advance in the approach to NPS in pre-dementia populations. Incorporating the MBI-C into case selection, which is free and easy to administer, may better identify those at risk, and those with preclinical or prodromal illness of pre-dementia patients. This group may then be worked up for pre-dementia markers, increasing the efficiency of clinical trial recruitment, and decreasing the cost (Mortby et al., 2018a).

## Conclusion and Future Directions

In this mini-review, we found that the present [^18^F]FDG PET studies are consistent with findings from functional connectivity studies that implicate dysfunctions in key regions of the SN and DMN in different subsyndromes of NPS in AD, further supporting NPS as early clinical manifestations of metabolic dysfunctions in regions susceptible to AD pathophysiology.

While the metabolic correlates of NPS are widely studied in AD dementia, studies on the predictive role of NPS in determining subsequent metabolic decline in preclinical AD remained limited. One possible reason could be the lack of a diagnostic tool designed to identify sustained NPS of later-life onset in non-demented persons as an early presentation of neurodegenerative disease. In this regard, the recently proposed MBI criteria enables the systematic study of NPS in cognitively normal individuals using a common language which is explicit with respect to cognitive status, and facilitating the differentiation between psychiatric disorders and NPS in preclinical and prodromal AD. Despite promising early findings, further research is needed to test the reliability and validity of the MBI criteria, to quantify the risk of late-onset NPS and incident dementia, and to validate the MBI-C in a wider population using different modes of administration and languages.

Recent longitudinal studies show that NPS are common in cognitively intact individuals and predict cognitive decline (Burhanullah et al., 2019; Wise et al., 2019). Therefore, individuals with MBI form an important clinical and research population for AD studies. Future research should combine [^18^F]FDG PET with functional studies and AD biomarkers, such as amyloid and tau, and should focus on the association of MBI with AD-related neurodegeneration, functional changes, and metabolic dysfunction. This will provide insight into the neurobiological basis of NPS in early AD, elucidate the role of MBI in the early detection of incident AD dementia, and facilitate the incorporation of MBI in the selection of individuals at risk for AD dementia for observational and clinical trials, especially in centers lacking access to AD biomarkers. Ultimately, the use of MBI in clinical practice to identify individuals with early presentation of AD may provide a window of opportunity to provide early interventions may alter disease course, delay the onset of dementia, and improve functional and cognitive outcomes.

## Author Contributions

KN did the study concept and design, compose table, and manuscript draft. HC did the study concept and design, compose table, and manuscript draft. PR-N did the study concept and manuscript draft. NK did the study concept and manuscript draft. ZI did the critical review of manuscript. SG did the study concept and design and critical review of manuscript for intellectual content.

## Funding

Our research is funded by the Canadian Institutes for Health Research.

## Conflict of Interest

The authors declare that the submitted work was not carried out in the presence of any personal, professional or financial relationships that could potentially be construed as a conflict of interest.
